# Study of the effect of antiviral therapy on homocysteinemia in hepatitis C virus- infected patients

**DOI:** 10.1186/1471-230X-12-117

**Published:** 2012-08-28

**Authors:** Mubin Mustafa, Sofia Hussain, Saleem Qureshi, Salman Akbar Malik, Ali Raza Kazmi, Muhammad Naeem

**Affiliations:** 1 Department of Biochemistry, Faculty of Biological Sciences, Quaid-i-Azam University, Islamabad, Pakistan; 2 Department of Biotechnology, Faculty of Biological Sciences, Quaid-i-Azam University, Islamabad, 45320, Pakistan; 3 Khan Research Laboratories (KRL) Hospital, Islamabad, Pakistan

**Keywords:** Hepatitis C virus, Infection, Interferon, Ribavirin, Homocysteine

## Abstract

**Background:**

Hepatitis C virus (HCV) infection is one of the leading causes of chronic liver disease (CLD). About 80% of those exposed to the virus develop a chronic infection. Hyperhomocysteinemia, which is an independent risk factor for atherosclerotic vascular disease and thromboembolism, may develop in HCV-infected patients although altered alanine amino transferase (ALT) enzyme levels are generally associated with damage to liver cells. The gold standard therapy for chronic hepatitis C patients is pegylated interferon combined with an anti-viral drug (ribavirin). The current study aimed to investigate the effect of antiviral therapy on plasma homocysteine (Hcy) levels in HCV patients in addition to other parameters.

**Methods:**

532 HCV-infected patients and 70 healthy controls were recruited for the study. All patients were subjected to laboratory investigations including HCV-RNA levels, complete blood cell counts, serum levels of homocysteine, ALT, alkaline phosphatase (ALP), lipid profile and liver ultrasonographic examination. The outcome of treatment with pegylated interferon α plus ribavirin treatment and sustained virologic response (SVR) was determined 6–9 months post-therapy.

**Results:**

Hyperhomocysteinemia was found in 91.35% of HCV-infected patients. The difference in plasma Hcy concentrations reached statistical significance between the patient and control groups. ALT, cholesterol and triglycerides (TGs) levels were found higher than normal in the patients group. After receiving a combined therapy for 24 weeks, 43.66% patients showed an SVR (responders); 30.98% patients were non-responders while 25.35% patients initially responded to therapy but again retrieved positive status of HCV infection six months post-therapy (relapse-cirrhotic patients). The mean levels of plasma Hcy, ALT and ALP were significantly reduced in responders within 10 weeks of therapy when compared with non-responders and relapse-cirrhotic patients.

**Conclusion:**

Elevated homocysteine levels in serum due to HCV infection can be reduced to normal range with the standard interferon α plus ribavirin treatment. This study highlights the significance of the measurement of serum homocysteine levels in the diagnosis and monitoring of HCV infection treatment in addition to other laboratory parameters.

## Background

Chronic infection with hepatitis C virus (HCV) is one of the leading causes of chronic liver disease; about 170 million people worldwide are estimated to be infected. Hepatitis C virus infection causes acute symptoms in only 15% of patients exposed to HCV infection while about 80% patients develop chronic infection [1]. Chronic hepatitis C results in formation of high levels of free radicals in the liver cells, which put serious oxidative stress depleting protective antioxidants and eventually kill the liver cells. Chronic hepatitis C infection progresses very slowly and is marked by episodes of acute hepatitis characterized by liver inflammation and elevated ALT levels. A hepatitis screen is recommended for patients whereby the disease can be diagnosed by the presence of antibodies for hepatitis C or by the direct presence of the virus or viral products in the blood [2]. Serum levels of liver enzymes such as alanine aminotransferase (ALT), aspartate aminotransferase (AST) and alkaline phosphatase (ALP) are analyzed to estimate damage caused to liver. Elevated ALT, AST & ALP are associated with varying degrees of damage to the liver cells [3].

Homocysteine levels are also altered in chronic liver disease. Homocysteine is a sulphur-containing amino acid belonging to the group of intracellular thiols. Numerous clinical and epidemiological studies have reported that elevated plasma homocysteine concentrations reflect impaired cellular metabolism [4] and may be considered as an independent risk factor for atherosclerotic vascular disease and thromboembolism [5]. Experimental data in transgenic mice deficient in homocysteine metabolism enzymes have shown the presence of severe liver steatosis with occasional steatohepatitis. In human beings, many studies have found a correlation between homocysteine and steatosis. Hyperhomocysteinemia may result from defects in homocysteine-metabolizing genes; vitamin B_6_, B_12_, or folate deficiencies resulting from nutritional conditions; or chronic alcohol consumption [6].

Homocysteine is mainly synthesized and metabolized in the liver, since metabolism of majority of dietary methionine occurs in this organ, where about 85% of the whole body capacity for transmethylation resides. Therefore, genes involved in methionine and homocysteine metabolism are expressed in a specific pattern in the liver [7]. Homocysteine is formed as an intermediate in methionine metabolism; therefore, impaired liver function leads to altered methionine and homocysteine metabolism [8].

The gold standard therapy for chronic hepatitis C virus-infected patients is a combined therapy with interferon (pegylated interferon) and an anti-viral drug (ribavirin). The first trial of interferon as therapy for chronic non-A, non-B hepatitis was reported in 1986. A sustained virologic response (SVR; clearance of serum HCV-RNA after six months of therapy) results from combined therapy with both pegylated interferon and ribavirin in 28% to 50% of patients with genotype 1, while in patients with genotype 2, sustained response rates are higher (76% to 82%) [9,10]. The recommended duration of treatment for HCV genotypes 2 and 3 is 24 weeks and for genotype 1 is 48 weeks. Sustained virologic response is usually accompanied by a return to normal serum ALT levels and improvement in inflammation within the liver. Apparently, the usual effect of interferon in patients with chronic hepatitis C who respond to this therapy is viral suppression but not eradication or cure. The genotype and level of viremia are important factors that affect the initial and long-term response to this therapy. The optimal treatment for non-responders and relapsers is not well established. However, it is expected that a minority of non-responders (6% to 12%) may respond to a second course of pegylated interferon and ribavirin [11].

The present study was conducted in Pakistan where more than 10 million people are living with hepatitis C virus with high morbidity and mortality. HCV prevalence is moderate in the general population (adults: 4.95% ± 0.53%; paediatric population: 1.72% ± 0.24%; young population: 3.64% ± 0.31%) but very high in injecting drug users (57% ± 17.7%) and multitransfused patients (48.67% ± 1.75%). The most prevalent genotype in Pakistan is 3a [12]. The aim of the current study was to evaluate the effect of combined antiviral therapy (interferon and ribavirin) on the concentrations of plasma homocysteine in HCV patients.

## Methods

### Selection of subjects

Approval for the study was obtained from Quaid-i-Azam University Institutional Review Board. Informed consent was obtained from all subjects who participated in the study. All the patients were positive for hepatitis C virus infection while the control subjects were all healthy. The patients were recruited from the Khan Research Laboratories (KRL) hospital Islamabad. The patients with hepatitis B virus infection, schistosomiasis, chronic parenchymal and obstructive renal diseases and alcohol abuse were excluded from the study. 532 patients (20–68 years; 272 males, 260 females) with hepatitis C virus infection (Group I) and 70 healthy control subjects (18–55 years; 42 males, 28 females; Group II) were included in the study. All the patients and controls were subjected to the following investigations: thorough history taking and physical examination, laboratory tests including complete blood cell counts (CBC), liver enzymes (ALT, AST & ALP), lipid profile (serum cholesterol, triglycerides, HDL, LDL), viral markers (PCR for HCV-RNA for those who tested positive for HCV-Ab), and plasma homocysteine concentration. Abdominal ultrasound and liver biopsy were performed for all subjects to evaluate liver and exclude any renal parenchymal disease or obstructive uropathy.

### Treatment with interferon

The patients’ group was given interferon therapy: as a first line of defence all the patients received 3 million U of uniferon α-2B plus an antiviral drug ribazole (1000 to1200 mg/day) for at least 24 weeks. A second line of defence therapy (uniferon or pegasys plus ribazole) was given after 6 months of completion of first dose only to those patients who were either non-responders or relapsed after first line of defence therapy.

Serum levels of HCV RNA were determined during a follow-up period (6–9 months post-treatment) with use of a reverse-transcriptase-PCR assay. All the patients were again subjected to thorough physical examination, lab tests, liver biopsy and abdominal ultrasound through and after the period of medication.

### Assessment of treatment efficacy

A sustained virologic response defined by the detectable levels of HCV RNA in serum, 24 weeks after the end of treatment was considered as primary end point. The absence of the detectable levels of HCV RNA in serum at the end of therapy and the normalization of serum ALT levels were considered as secondary end points.

### Statistical analysis

All values were expressed as arithmetic mean ± SD. The difference of biochemical and other parameters between the control and patient groups before treatment was tested for statistical significance using *t*-test. For overall comparisons, one-way analysis of variance (ANOVA) was used. The difference in serum homocysteine and liver enzyme levels between differently responding-patient groups after interferon treatment was investigated by Tukey’s honestly significant difference (HSD) test. P-value ≤ 0.0001 was considered extremely statistically significant, ≤ 0.001 as very statistically significant and ≤ 0.01 as statistically significant.

## Results

### Hyperhomocysteinemia and serum ALT levels before interferon treatment

**Figure 1:**
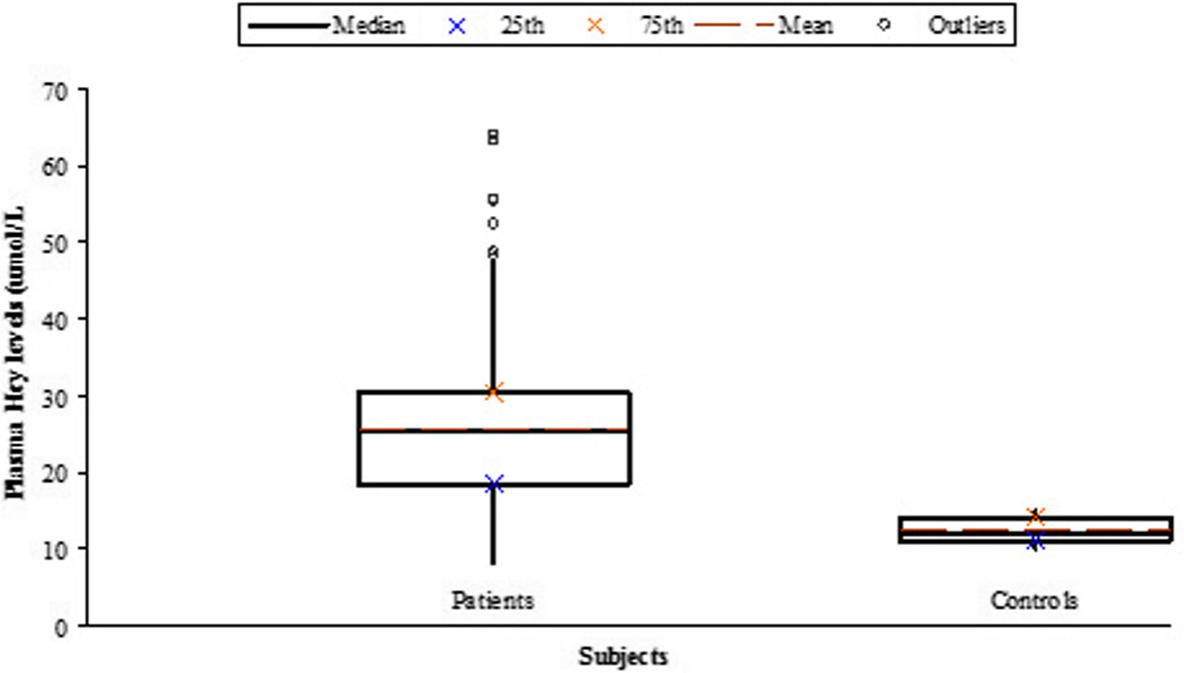
Plasma homocysteine (Hcy) levels in patients and controls.

We studied 532 HCV-infected patients (272 males; 260 females) and 70 healthy individuals (42 males; 28 females) were enrolled as controls. Hyperhomocysteinemia was defined as plasma Hcy level > 15 μmol/L. Hyperhomocysteinemia was observed in 486 patients (253 males; 233 females). The mean level of plasma Hcy was significantly higher in the patients group (25.66 + 8.89) when compared to the control group (12.36 + 1.64). The difference in plasma Hcy concentrations reached statistical significance between the control and patient groups (Table 1; Figure 1). ALT levels were found higher than normal in the patients’ group (110.47 ± 128.85). Cholesterol and triglyceride levels were also elevated in the patients.

**Table 1:** Base-line characteristics of controls and patients before antiviral treatment

**Laboratory parameters**	**Reference values**	**Control Group (n = 70)**	**Patients Group (n = 532)**	**P**
Hb (g/dl)	M:13.5-18.0; F:11.5-16.5	14.19 ± 1.65	13.01 ± 1.98	<0.0001***
PLT (*100)	150-450*100	276.64 ± 75.31	545.79 ± 85.38	<0.0001***
TLC	4000-11000	6.14 ± 1.53	5.62 ± 2.36	=0.01*
ESR (mm/hr)	M:0–10; F:0-15	13.18 ± 1.47	37.63 ± 22.34	<0.0001***
ALT (U/L)	M:0–40; F:0-37	33.2 ± 7.45	110.47 ± 128.85	<0.0001***
ALP (U/L)	60-306	214.61 ± 64.23	259.64 ± 64.07	<0.0001***
Cholesterol (mmol/L)	Normal: 3.4-5.2; Borderline high: 5.2-6.2; High: >6.22	4.67 ± 0.36	5.6 ± 0.76	<0.0001***
HDL (mmol/L)	0.8-1.7	1.11 ± 0.16	1.30 ± 0.44	<0.0001***
LDL (mmol/L)	<3.8	2.92 ± 0.29	3.27 ± 0.72	<0.0001***
TGs (mmol/L)	0.4-1.9	1.31 ± 0.49	2.10 ± 0.70	<0.0001***
Hcy (μmol/L)	5-15	12.36 ± 1.64	25.66 ± 8.89	<0.0001***

***: extremely statistically significant; **: very statistically significant; *: statistically significant; +: statistically non-significant. Hb: hemoglobin; PLT: platelets; TLC: total leukocyte count; ESR: erythrocyte sedimentation rate; ALT: alanine amino transferase; ALP: alkaline phosphatase; HDL: high density lipids; LDL: low density lipids; TGs: triglycerides; Hcy: homocysteine.

### Hyperhomocysteinemia and serum ALT levels after interferon treatment

On the basis of the effect of combined antiviral therapy given, the individuals in the patients’ group who were available for follow-up after treatment (n = 355; 177 patients were lost to follow-up after treatment) were divided into three subgroups as responders (group I: n = 155; 43.66%), non-responders (group II: n = 110; 30.98%) and relapse-cirrhotic patients (group III: n = 90; 25.35%). Responders were the group of patients in which HCV RNA was found below the detection limit or turns to normal after completion of 24 weeks of interferon therapy; non-responders were those HCV patients who did not show a response to either first line or the subsequent second line of combined therapy; the third group (relapse-cirrhotic patients) was found normal after completion of the interferon therapy, however, regained HCV positive status after 6 months of completion of therapy. The mean level of plasma Hcy was significantly reduced in responders (group I; 19.33 ± 2.43), when compared to non-responders (group II; 44.04 ± 9.32) and relapse-cirrhotic patients (group III; 56.11 ± 14.63). The difference in plasma Hcy concentrations reached statistical significance among the three studied groups (Table 2).

**Table 2:** Comparison of laboratory parameters between different patient groups after antiviral treatment

**Biochemical Parameters**	**Groups**			**Overall**	**Comparisons between groups**		
	**Group I Responders (n = 155)**	**Group II Non-responders (n = 110)**	**Group III Relapse-cirrhotics (n = 90)**	***P***	**I & II**	**I &III**	**II & III**
Hb (g/dl)	14.56194 ± 1.197414	18.1790909 ± 1.002574251	11.22778 ± 1.300564	<0.0001***	<0.01*	<0.01*	<0.01*
PLT (*100)	237.7032 ± 63.00476	130.724771 ± 36.55282113	122.9111 ± 35.38286	0.6^+^	-	-	-
TLC	6.129032 ± 1.145968	12.5654545 ± 2.160463608	6.246667 ± 1.324887	<0.0001***	<0.01*	NS	<0.01*
ESR (mm/hr)	19.63226 ± 7.286368	40.6818182 ± 22.31177408	29.93333 ± 10.32592	<0.0001***	<0.01*	<0.01*	<0.01*
ALT1 (U/L)	118.5484 ± 36.21046	76.0727273 ± 23.96354613	102 ± 68.31355	<0.0001***	<0.01*	<0.05*	<0.01*
ALT2 (U/L)	72.77419 ± 17.2674	121.154545 ± 32.52499007	82.44444 ± 56.79178	<0.0001***	<0.01*	NS	<0.01*
ALT3 (U/L)	41.42581 ± 9.053914	181.327273 ± 48.44122687	115.2889 ± 64.24326	<0.0001***	<0.01*	<0.01*	<0.01*
ALP1 (U/L)	336.2516 ± 20.15414	304.045455 ± 12.09623974	349.9111 ± 25.01624	<0.0001***	<0.01*	<0.01*	<0.01*
ALP2 (U/L)	251.6258 ± 24.00545	340.227273 ± 10.83035074	324.8778 ± 24.07938	<0.0001***	<0.01*	<0.01*	<0.01*
ALP3 (U/L)	170.9484 ± 28.92799	380.763636 ± 12.85267778	365.7222 ± 20.64849	<0.0001***	<0.01*	<0.01*	<0.01*
CHOL (mmol/L)	4.643226 ± 0.535738	5.68090909 ± 0.426742613	5.631111 ± 0.777007	<0.0001***	<0.01*	<0.01*	NS
HDL (mmol/L)	1.343226 ± 0.495305	1.29454545 ± 0.297955222	1.357778 ± 0.444436	0.53^+^	-	-	-
LDL (mmol/L)	3.343226 ± 0.531846	4.25636364 ± 0.408516509	3.153333 ± 1.103233	<0.0001***	<0.01*	NS	<0.01*
TGs (mmol/L)	1.93871 ± 0.567792	2.27454545 ± 0.586614664	2.026667 ± 0.592424	<0.0001***	<0.01*	<0.01*	<0.01*
Hcy (μmol/L)	19.33652 ± 2.434538	44.0485455 ± 9.32508345	56.11422 ± 14.63146	<0.0001***	<0.01*	<0.01*	<0.01*

***: extremely statistically significant; **: very statistically significant; *: statistically significant; +: statistically non-significant. Hb: hemoglobin; PLT: platelets; TLC: total leukocyte count; ESR: erythrocyte sedimentation rate; ALT1, ALT2 & ALT3: alanine amino transferase levels before, during and after therapy, respectively; ALP1, ALP2 & ALP3: alkaline phosphatase levels before, during and after therapy, respectively; CHOL: cholesterol; HDL: high density lipids; LDL: low density lipids; TGs: triglycerides; Hcy: homocysteine.

**Figure 2:**
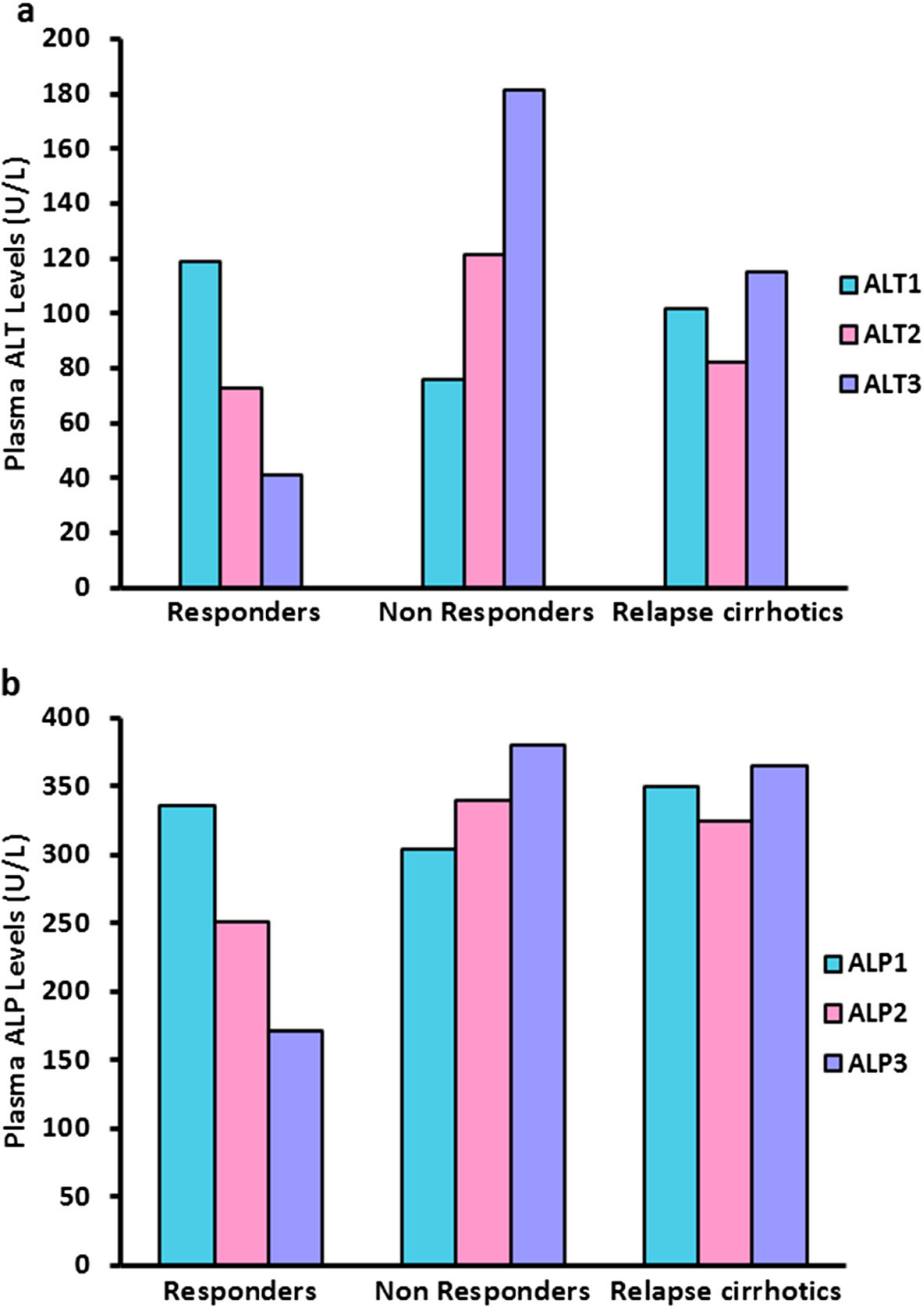
**a) Comparative plasma ALT levels in different patient groups at different stages of interferon therapy.b)** Comparative plasma ALP levels in different patient groups at different stages of interferon therapy.

At the start of the treatment, the ALT and ALP levels were higher than normal in the three treatment groups. After therapy, these levels were normalized in responders (group I) While non responders did not show any decline. The ALT and ALP levels were reduced in relapse-cirrhotic patients (group III) following the therapy but again elevated even after treated with a second line of therapy (Table 2; Figure 2).

## Discussion

Our results indicated that plasma homocysteine levels were significantly elevated in HCV-infected patients in both sexes compared with control values. These findings are in accordance with the results of Taha et al. who observed elevated plasma homocysteine levels in patients with liver cirrhosis secondary to hepatitis C virus infection [4]. Garacia et al. attributed this condition to reduced expression of genes involved in Hcy metabolism. The degree of reduced expression of these genes was related to the severity of liver disease [6]. The hyperhomocysteinemia is known as atherogenic and thrombotic risk factor for cardiovascular disease. It might also be a risk factor for cirrhotic patients but the direct effect of Hcy on liver injury is not well known [13]. Hyperhomosysteinemia was correlated with elevated levels of ALT, ALP, TGs and cholesterol in the current study. This might be related to progression of liver injury. Some other studies have also reported a correlated elevation of plasma Hcy levels with ALT, ALP, TGs and cholesterol [14,15]. It is evident that homocysteine-induced endoplasmic reticulum stress leaves a dysregulated endogenous sterol response pathway, which leads to increased hepatic biosynthesis and uptake of cholesterol and triglycerides [16].

This study addressed the efficacy of a combination therapy of interferon with an antiviral drug for the treatment of hepatitis C virus infection and recovery of Hcy, ALT and ALP levels to normal. Interferon-α (IFN-α) is a cytokine having multiple biological functions, which includes antiviral and immunomodulatory activities [17], and is commonly used for the treatment of patients with chronic HCV infection. It has been evident from the results of recent clinical reports that IFN-α treatment is effective in decreasing serum ALT levels, reducing and eliminating serum HCV RNA, and improving liver histology in patients with chronic hepatitis C [18-22]. We performed a comparative analysis of sustained virologic response (clearance of serum HCV-RNA after six months of therapy), homocysteinemia, serum ALT and ALP levels in HCV-infected patients. Consequently, a positive response was observed in 43.66% patients as indicated by undetectable serum HCV RNA (SVR), normal homocysteine, ALT and ALP levels (referred to as responders). Other (25.35%) patients shown positive response to therapy initially but again retrieved HCV positive status (relapse-cirrhotic patients). The therapy remained ineffective in 30.98% patients (non-responders). Increased homocysteine levels (>16 μmol/L) represent a factor associated with a lower rate of SVR. Association between high homocysteine levels and increased oxidative stress on cells partially explains this effect [23]. It has been reported that homocysteine transsulfuration through cystathionine-β-synthase (CBS) activity as well as its remethylation through betaine-dependent methyltransferase (BHMT) activity are restricted to the liver [24]. Hence, an increase in homocysteine levels could be the result of reduced activities of these enzymes in patients with liver diseases, as reported in the present study.

In the current study, anti-viral treatment for 24 weeks was sufficient in HCV-infected patients to achieve SVR, since a second line of therapy for prolonged periods was still inefficient to reduce viremia in relapse-cirrhotic patients. Even shorter periods of treatment might also be sufficient in patients whom serum levels of HCV RNA quickly become undetectable. These findings are in line with the results of Jaeckel et al. [15]. The present study also confirmed that the combination treatment (standard interferon α plus ribavirin) can eliminate virus in about half of HCV-infected patients [25].

As mentioned earlier that genotype 3a is the most prevalent HCV genotype in the local population, we can presume that findings of the current study predominantly correlate with genotype 3a and should not be extrapolated to other genotypes without further research.

## Conclusion

We studied the effect of standard antiviral therapy (interferon α plus ribavirin) on different laboratory parameters in HCV patients in Pakistan. Liver enzymes and homocysteine levels, which are elevated in the serum of HCV patients beyond reference values (indicated for normal individuals), were reduced to normal limits after the treatment. However, the overall efficacy of standard antivirus therapy in multigenotype subjects remained low (~43%) that warns the need of more efficient drugs with better efficacy. The study strengthens the evidence that supports the significance of serum homocysteine levels in the diagnosis and monitoring of HCV infection treatment in addition to other laboratory parameters. Further studies in different HCV genotypes are suggested to establish serum homocysteine level as a biomarker.

## Competing interests

The authors declare that they have no competing interests.

## Authors’ contributions

MM planned study & performed bench work. SH & MN analyzed the data and prepared the manuscript. SQ, ARK & SAM planned and supervised the research work. All authors read and approved the final manuscript.

## Pre-publication history

The pre-publication history for this paper can be accessed here:

http://www.biomedcentral.com/1471-230X/12/117/prepub
